# Association Between Greenness Surrounding Schools and Kindergartens and Attention-Deficit/Hyperactivity Disorder in Children in China

**DOI:** 10.1001/jamanetworkopen.2019.17862

**Published:** 2019-12-18

**Authors:** Bo-Yi Yang, Xiao-Wen Zeng, Iana Markevych, Michael S. Bloom, Joachim Heinrich, Luke D. Knibbs, Shyamali C. Dharmage, Shao Lin, Pasi Jalava, Yuming Guo, Bin Jalaludin, Lidia Morawska, Yang Zhou, Li-Wen Hu, Hong-Yao Yu, Yunjiang Yu, Guang-Hui Dong

**Affiliations:** 1 Guangdong Provincial Engineering Technology Research Center of Environmental and Health Risk Assessment, Department of Occupational and Environmental Health, Sun Yat-sen University, Guangzhou, China; 2 Institute and Clinic for Occupational, Social and Environmental Medicine, University Hospital, Ludwig Maximilian University of Munich, Munich, Germany; 3 Institute of Epidemiology, Helmholtz Zentrum München–German Research Center for Environmental Health, Neuherberg, Germany; 4 Division of Metabolic and Nutritional Medicine, Dr von Hauner Children’s Hospital, Ludwig Maximilian University of Munich, Munich, Germany; 5 Department of Environmental Health Sciences, University at Albany, State University of New York, Rensselaer; 6 Department of Epidemiology and Biostatics, University at Albany, State University of New York, Rensselaer; 7 Comprehensive Pneumology Center Munich, German Center for Lung Research, Munich, Germany; 8 School of Public Health, The University of Queensland, Herston, Queensland, Australia; 9 Allergy and Lung Health Unit, Centre for Epidemiology and Biostatistics, School of Population and Global Health, The University of Melbourne, Melbourne, Victoria, Australia; 10 Murdoch Children Research Institute, Melbourne, Victoria, Australia; 11 Department of Environmental and Biological Sciences, University of Eastern Finland, Kuopio, Finland; 12 Department of Epidemiology and Preventive Medicine, School of Public Health and Preventive Medicine, Monash University, Melbourne, Victoria, Australia; 13 Centre for Air Quality and Health Research and Evaluation, Glebe, New South Wales, Australia; 14 Population Health, South Western Sydney Local Health District, Liverpool, New South Wales, Australia; 15 Ingham Institute for Applied Medical Research, Liverpool, New South Wales, Australia; 16 School of Public Health and Community Medicine, The University of New South Wales, Kensington, New South Wales, Australia; 17 International Laboratory for Air Quality and Health, Brisbane, Queensland University of Technology, Queensland, Australia; 18 Science and Engineering Faculty, Queensland University of Technology, Brisbane, Queensland, Australia; 19 State Environmental Protection Key Laboratory of Environmental Pollution Health Risk Assessment, South China Institute of Environmental Sciences, Ministry of Environmental Protection, Guangzhou, China

## Abstract

**Importance:**

Few studies have investigated the association between greenness and childhood attention-deficit/hyperactivity disorder (ADHD).

**Objective:**

To evaluate the association between greenness surrounding schools or kindergartens and symptoms of ADHD in children.

**Design, Setting, and Participants:**

This population-based cross-sectional study was performed between April 2012 and January 2013 in 7 cities in northeastern China. This analysis included 59 754 children (aged 2-17 years) from 94 schools and kindergartens, who had resided in the study area for 2 years or longer. Data were analyzed from April 15, 2019, to October 10, 2019.

**Exposures:**

Greenness surrounding each child’s school or kindergarten was estimated using 2 satellite image–derived vegetation indexes: the normalized difference vegetation index and the soil-adjusted vegetation index.

**Main Outcomes and Measures:**

*Diagnostic and Statistical Manual of Mental Disorders* (Fourth Edition) scales were used to measure ADHD symptoms (9 inattention symptoms and 9 hyperactivity-impulsivity symptoms). Parents or guardians rated the frequency of each of 18 ADHD symptoms during the preceding 6 months. Children with 6 or more symptoms of either inattention or hyperactivity-impulsivity were defined as having ADHD symptoms. Generalized linear mixed models were applied to estimate the association between greenness and ADHD symptoms.

**Results:**

The mean (SD) age of the 59 754 study participants was 10.3 (3.6) years, and 29 494 (49.4%) were girls. A total of 2566 participants (4.3%) had ADHD symptoms. Greenness levels differed substantially across schools and kindergartens. The normalized difference vegetation index within 500 m of a school or kindergarten ranged from −0.09 to 0.77. Greater greenness levels were associated with lower odds of ADHD symptoms. In covariate-adjusted models, a 0.1-unit increase in normalized difference vegetation index or soil-adjusted vegetation index within 500 m of a school or kindergarten was significantly associated with lower odds of ADHD symptoms (odds ratios, 0.87 [95% CI, 0.83-0.91] and 0.80 [95% CI, 0.74-0.86], respectively; *P* < .001 for both). The associations were robust in a series of sensitivity analyses.

**Conclusions and Relevance:**

These findings suggest that there may be a beneficial association between school-based greenness and ADHD symptoms in Chinese children. Future longitudinal and mechanistic studies are needed to confirm the findings of this cross-sectional analysis and further explore potential mechanisms of this association.

## Key Points

**Question:**

Is greenness surrounding schools and kindergartens associated with symptoms of attention-deficit/hyperactivity disorder among children?

**Findings:**

In this large cross-sectional study of 59 754 Chinese children, attendance at schools or kindergartens in greener areas was associated with lower odds of having attention-deficit/hyperactivity disorder symptoms.

**Meaning:**

These findings may be useful for policy makers and health care professionals to develop strategies (eg, planning for green spaces around schools and kindergartens) to mitigate the attention-deficit/hyperactivity disorder burden.

## Teaser

This cross-sectional study evaluates the association between greenness surrounding schools or kindergartens and symptoms of attention-deficit/hyperactivity disorder among Chinese children.

## Introduction

Attention-deficit/hyperactivity disorder (ADHD) is among the most common neurobehavioral conditions in childhood. Globally, approximately 7.2% of children are estimated to have ADHD.^1^ China also has a substantial number of children with ADHD. In a recent meta-analysis^2^ of 67 Chinese studies of children, the prevalence of ADHD reached 6.3%. Prior evidence suggests that ADHD has a range of negative outcomes on individuals, as well as economic burdens on families and society.^3,4^ Because environmental factors are associated with ADHD,^5^ identifying such factors may be useful for reducing ADHD symptoms, especially considering that environmental factors are generally modifiable.

Accumulating evidence indicates that living in greener areas is associated with many beneficial health outcomes.^6^ The underlying mechanisms include reducing harmful exposures, such as air pollution and noise, encouraging physical activity, mitigating mental stress, and enriching microbial diversity.^6^ Several previous epidemiological studies^7,8,9,10,11,12,13,14^ investigated the association between greenness and ADHD (or ADHD symptoms) in all children and with ADHD severity in children with a diagnosis of ADHD, but the results were inconsistent (eTable 1 in the Supplement). In addition, most of the published studies focused on residential greenness.^7,8,9,11,12,13,14^ Children spend long periods at school, studying and participating in outdoor activities; thus, they may have more opportunities to use school green spaces than residential green spaces. To date, we are aware of only 1 prior study^10^ investigating the associations of school greenness with ADHD symptoms, and it was conducted in a developed Western nation. No such evidence is available from developing nations like China, where students spend more time at school because of the heavy school workload.

To address this data gap, we conducted a large population-based study in northeastern China to evaluate associations between greenness surrounding school and kindergarten centroids and ADHD symptoms in children aged 2 to 17 years. Our hypothesis was that greater school or kindergarten greenness would be associated with a lower odds of having ADHD symptoms.

## Methods

### Participants and Procedures

Between April 2012 and January 2013, we completed the Seven Northeastern Cities Study in Liaoning Province, northeastern China. A 3-stage stratified clustering sampling strategy was used to select study participants (Figure). First, we randomly selected 7 of 14 provincial cities in Liaoning Province. Second, from each district (24 districts in total), we randomly selected 1 or 2 kindergartens, 1 or 2 elementary schools, and 1 or 2 middle schools, resulting in a total of 94 schools and kindergartens. Third, all students in the 94 schools were eligible if they had lived at their current address for 2 years or longer. We obtained permission from each school or kindergarten’s principal and then provided classroom teachers with information packets that included study descriptions, consent forms, and questionnaires, to distribute to students’ parents or guardians. We carefully emphasized the noncompulsory nature of participation to classroom teachers to ensure voluntary participation by parents or guardians. We also invited consenting parents or guardians to a study information session. Study questionnaires were completed by parents or guardians at the time of the information session or at home, in which case the student delivered the completed document to the classroom teacher. Study questionnaires included queries related to sociodemographic information, lifestyles, parental socioeconomic status, and measurement scales for ADHD symptoms.

**Figure.**
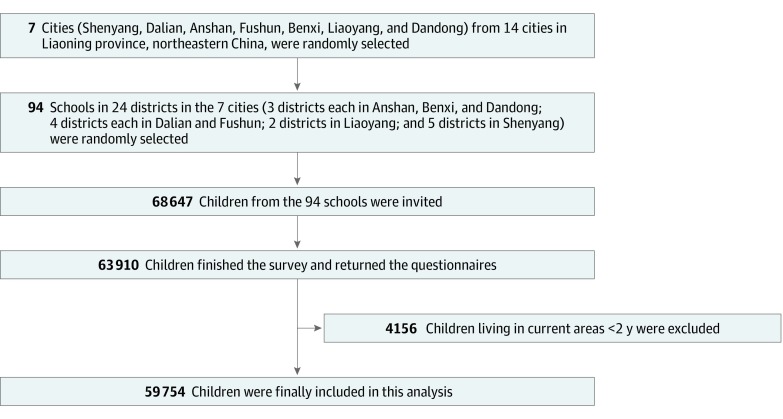
Sampling Strategy for the Seven Northeastern Cities Study Flowchart shows sampling strategy for children included in this study.

 All participants’ parents or guardians gave written informed consent. The Human Studies Ethics Committee of Sun Yat-Sen University reviewed and approved the study protocol. This study follows the Strengthening the Reporting of Observational Studies in Epidemiology (STROBE) reporting guideline for cross-sectional studies.

### ADHD Symptoms

We used *Diagnostic and Statistical Manual of Mental Disorders* (Fourth Edition) (*DSM-IV*) ADHD scales to measure ADHD symptoms.^15^ Participants’ parents or guardians completed the ADHD *DSM-IV* survey, which includes 9 inattention symptoms and 9 hyperactivity-impulsivity symptoms. Respondents rated the frequency of each ADHD symptoms in the preceding 6 months on a 4-point scale: never or rare, 0; sometimes, 1; often, 2; or very often, 3. We used the Chinese version of the *DSM-IV* ADHD scale, which has been demonstrated to be valid and reliable for the Chinese population.^16^ Consistent with *DSM-IV* criteria, children presenting with 6 or more symptoms of either inattention or hyperactivity-impulsivity were defined as having ADHD symptoms. The symptoms of ADHD can be classified into 3 subtypes: predominantly inattention, predominantly hyperactivity-impulsivity, and both inattention and hyperactivity-impulsivity.

To assess the robustness of our outcome assessment strategy, we also used the 10-item Conners Abbreviated Symptom Questionnaire (C-ASQ) to identify ADHD symptoms.^17^ The C-ASQ measures ADHD symptoms on a 4-point scale: never or rare, 0; sometimes, 1; often, 2; or very often, 3. The C-ASQ scores for ADHD symptoms thus range from 0 to 30, and a score greater than 15 was defined as the presence of ADHD symptoms.^17^ The C-ASQ is derived from the Revised Conners Parent Rating Scale and has been translated and validated for use in Chinese population.^18^

### Greenness Surrounding School or Kindergarten

We assessed greenness surrounding schools and kindergartens by 2 vegetation indices: the normalized difference vegetation index (NDVI)^19^ and the soil-adjusted vegetation index (SAVI).^20^ Both indices were derived from Landsat 5 Thematic Mapper satellite images obtained at a resolution of 30 m × 30 m. The derivation of NDVI is based on land surface reflectance of visible (red) and near-infrared parts of the spectrum, and SAVI also incorporates a correction factor to minimize the impact of soil background. Both NDVI and SAVI values range from −1 to 1, and although values do not map to specific types of land cover, higher values represent more greenness (eg, leaves and grasses), negative values represent water bodies, and values close to 0 generally correspond to barren areas (eg, rock). We downloaded 2 cloud-free satellite images taken in August 2010, the greenest month in northeastern China and the time closest to health data collection. School and kindergarten greenness was estimated as the mean NDVI and SAVI values in buffers of 100 m, 500 m, and 1000 m around the centroid of each school or kindergarten. Both NDVI and SAVI were calculated using ArcGIS visualization software version 10.4 (ESRI).

### Confounders

We used a questionnaire to collect data on the following variables: children’s age (years), children’s sex (male vs female), parental education level (defined as the highest level of education completed by either parent: completed high school or higher, some high school, middle school, and primary school), annual household income level (≤9999 Yuan, 10 000-29 999 Yuan, 30 000-100 000 Yuan, and >100 000 Yuan; as of November 25, 2019, to convert Yuan to US dollars, multiply by 0.14), type of home district (residential, industrial, and business), dog ownership (yes or no), cigarette smoking during pregnancy (yes vs no), alcohol drinking during pregnancy (yes vs no), breastfeeding (yes [defined as maternal report of having mainly breastfed for ≥3 months] vs no [defined as maternal report of having mainly breastfed for <3 months]), preterm birth (yes [defined as <37 weeks’ gestation] vs no [defined ≥37 weeks’ gestation]), number of siblings (0 vs ≥1), and birth weight (grams). Then, we developed a directed acyclic graph (eFigure in the Supplement), informed by the existing literature and expert knowledge, to select a minimally sufficient set of covariates.^21^ The following variables were retained as confounders in multivariable regression models: children’s age,^22,23^ children’s sex,^24,25^ parental education level,^26,27^ household income level,^26,27^ type of home district,^26,27^ and dog ownership.^28^

### Statistical Analysis

As a result of the multilevel nature of the outcomes among children within schools or kindergartens, we used generalized linear mixed models to assess the association between greenness and ADHD symptoms. We incorporated school or kindergarten as a random effect and greenness metrics and covariates as fixed effects. Odds ratios (ORs) and 95% CIs per 0.1-unit greater NDVI and SAVI were estimated. Because of the generalized linear mixed models framework, the ORs are school or kindergarten specific and not population averaged. We estimated crude models, unadjusted regression models, and regression models adjusted for covariates selected using the directed acyclic graph (eFigure in the Supplement).

We performed several sensitivity analyses to evaluate the robustness of the results in our main effects models. First, we estimated NDVI and SAVI in buffers of 100 and 1000 m to assess the impact of exposure misclassification. We also categorized NDVI for 500 m (NDVI_500-m_) and SAVI for 500 m (SAVI_500-m_) into quartiles to check for nonlinear associations. We split ADHD into subtypes (ie, predominantly inattention, predominantly hyperactivity-impulsivity, and both inattention and hyperactivity-impulsivity) to compare the association of greenness with different subsymptoms of ADHD to assess specificity of the associations, and we also redefined ADHD using the C-ASQ to assess the effect of outcome misclassification. We generated regression models with additional adjustment for maternal smoking during pregnancy,^29^ maternal alcohol consumption during pregnancy,^30^ breastfeeding,^31^ preterm birth,^32^ birth weight,^33^ and number of siblings^34^ to assess those associations with ADHD symptoms. Finally, we excluded children with allergic diseases (defined as having physician-diagnosed eczema, allergic conjunctivitis, and/or asthma),^35^ congenital heart diseases,^36^ preterm birth,^32^ or low birth weight (defined as birth weight <2500 g)^33^ as a group potentially with greater baseline risk for ADHD.

Next, we tested whether children’s age and sex, household income, type of home district, and dog ownership modified the associations between greenness and ADHD symptoms. We incorporated interaction terms between greenness and modifiers into the adjusted regression models to test for interactions, and then stratified according to subgroups for interpretation.

Data were analyzed from April 15, 2019, to October 10, 2019. All statistical analyses, including χ^2^ test, Wilcoxon signed rank test, and likelihood ratio test, were conducted in SAS statistical software version 9.4 (SAS Institute). A 2-tailed *P* < .05 denoted statistical significance.

## Results

### Descriptive Statistics

A total of 68 647 children’s parents or guardians were invited to participate. Of those, 63 910 completed and returned a study questionnaire (response rate, 93.1%). We then excluded 4156 children who resided at their current address for less than 2 years. Finally, 59 754 children were included in this analysis.

Of the 59 754 children included in the analysis, 2566 (4.3%) had ADHD symptoms (Table 1). The mean (SD) age of the children was 10.3 (3.6) years, and 29 494 (49.4%) were girls. Compared with children without ADHD symptoms, those with ADHD symptoms were more likely to be boys (64.2% [1647] vs 50.0% [28 613]), aged 7 years or older (92.3% [2369] vs 83.2% [47 562]), born to parents with lower education (38.4% [986] vs 26.2% [14 982]) or lower income (66.5% [1705] vs 57.6% [32 924]), have 1 or more siblings (17.7% [454] vs 15.3% [8730]), born preterm (7.3% [188] vs 5.3% [3029]), born to a cigarette smoker (1.5% [39] vs 0.6% [328]) or alcohol drinker (1.7% [44] vs 0.7% [405]), and to live in industrial or business areas (14.0% [360] vs 12.2% [6974]). However, no significant difference was observed for breastfeeding (65.4% [1678] vs 66.6% [38 078]) and low birth weight (4.4% [112] vs 3.6% [2075]) between children with and without ADHD symptoms.

**Table 1.** Characteristics of the Study Participants According to ADHD Status

Characteristic	Participants, No. (%) (N = 59 754)		*P* Value
	Children With ADHD (n = 2566)	Children Without ADHD (n = 57 188)	
Age, y			
2-6	197 (7.7)	9626 (16.8)	<.001
7-17	2369 (92.3)	47 562 (83.2)	
Sex			
Male	1647 (64.2)	28 613 (50.0)	<.001
Female	919 (35.8)	28 575 (50.0)	
Parental education level, y			
≤9	986 (38.4)	14 982 (26.2)	<.001
>9	1580 (61.6)	42 206 (73.8)	
Annual household income, Yuan^a^			
≤30 000	1705 (66.5)	32 924 (57.6)	<.001
>30 000	861 (33.6)	24 264 (42.4)	
Type of home district			
Residential	2206 (86.0)	50 214 (87.8)	.01
Industrial or business	360 (14.0)	6974 (12.2)	
Dog ownership			
No	2377 (92.6)	54 003 (94.4)	<.001
Yes	189 (7.4)	3185 (5.6)	
Cigarette smoking during pregnancy			
No	2527 (98.5)	56 860 (99.4)	<.001
Yes	39 (1.5)	328 (0.6)	
Alcohol drinking during pregnancy			
No	2522 (98.3)	56 783 (99.3)	<.001
Yes	44 (1.7)	405 (0.7)	
Breastfeeding			
No	888 (34.6)	19 110 (33.4)	.21
Yes	1678 (65.4)	38 078 (66.6)	
No. of siblings			
0	2112 (82.3)	48 458 (84.7)	.001
≥1	454 (17.7)	8730 (15.3)	
Low birth weight			
No	2454 (95.6)	55 113 (96.4)	.05
Yes	112 (4.4)	2075 (3.6)	
Preterm birth			
No	2378 (92.7)	54 159 (94.7)	<.001
Yes	188 (7.3)	3029 (5.3)	
Normalized difference vegetation index			
Within 100 m of school or kindergarten, median (IQR)	0.26 (0.21-0.35)	0.27 (0.22-0.38)	<.001
Within 500 m of school or kindergarten, median (IQR)	0.27 (0.23-0.33)	0.30 (0.24-0.38)	<.001
Within 1000 m of school or kindergarten, median (IQR)	0.27 (0.23-0.33)	0.30 (0.24-0.36)	<.001
Soil-adjusted vegetation index			
Within 100 m of school or kindergarten, median (IQR)	0.14 (0.11-0.20)	0.16 (0.12-0.22)	<.001
Within 500 m of school or kindergarten, median (IQR)	0.15 (0.12-0.20)	0.17 (0.14-0.21)	<.001
Within 1000 m of school or kindergarten, median (IQR)	0.15 (0.13-0.19)	0.16 (0.14-0.20)	<.001

Abbreviations: ADHD, attention-deficit/hyperactivity disorder; IQR interquartile range.

^a^ As of November 25, 2019, to convert Yuan to US dollars, multiply by 0.14.

Greenness levels varied substantially across the schools and kindergartens (eg, NDVI_500-m_ values ranged from −0.09 to 0.77). The NDVI and SAVI values were positively correlated with each other (*r*, 0.70 to 0.99) (eTable 2 in the Supplement). Children with ADHD symptoms attended schools with lower greenness levels (median [interquartile range] NDVI_500-m_, 0.27 [0.23 to 0.33]; SAVI_500-m_, 0.15 [0.12 to 0.20]) than those without ADHD symptoms (median [interquartile range], NDVI_500-m_, 0.30 [0.24 to 0.38]; SAVI_500-m_, 0.17 [0.14 to 0.21]) (Table 1). Of note, the mean distance between school and home for the participants was 1 km (data not shown).

### Is School Greenness Associated With ADHD Symptoms?

In unadjusted models, we observed that higher greenness exposure was significantly associated with lower odds of ADHD symptoms (eg, per 0.1-unit greater NDVI_500-m_ and SAVI_500-m_, OR, 0.81 [95% CI, 0.76-0.86] and 0.72 [95% CI, 0.65-0.79], respectively; *P* < .001 for both) (Table 2). The associations were similar after adjusting for age, sex, parental education level, parental income level, type of home district, and dog ownership. For example, a 0.1-unit increase in NDVI_500-m_ and SAVI_500-m_ was significantly associated with a lower odds of ADHD symptoms (OR, 0.87 [95% CI, 0.83-0.91] and 0.80 [95% CI, 0.74-0.86], respectively; *P* < .001 for both).

**Table 2.** Associations Between 0.1-Unit Increase in Greenness Metrics and Attention-Deficit/Hyperactivity Disorder Symptoms

Greenness Metrics	Crude Model		Adjusted Model^a^	
	OR (95% CI)	*P* Value	OR (95% CI)	*P* Value
Normalized difference vegetation index				
Within 100 m of school or kindergarten	0.87 (0.82-0.92)	<.001	0.92 (0.89-0.97)	<.001
Within 500 m of school or kindergarten	0.81 (0.76-0.86)	<.001	0.87 (0.83-0.91)	<.001
Within 1000 m of school or kindergarten	0.79 (0.73-0.84)	<.001	0.84 (0.80-0.88)	<.001
Soil-adjusted vegetation index				
Within 100 m of school or kindergarten	0.81 (0.74-0.88)	<.001	0.90 (0.83-0.95)	<.001
Within 500 m of school or kindergarten	0.72 (0.65-0.79)	<.001	0.80 (0.74-0.86)	<.001
Within 1000 m of school or kindergarten	0.69 (0.62-0.77)	<.001	0.76 (0.70-0.82)	<.001

Abbreviation: OR, odds ratio.

^a^ Adjusted for age, sex, parental education level, household income level, type of home district, and dog ownership.

We repeated the analysis using NDVI and SAVI in 100-m and 1000-m buffers and found that the direction of the associations were consistent with those of the main analysis for a 500-m buffer (NDVI for 100 m, OR, 0.87 [95% CI, 0.82-0.92] in the crude model and 0.92 [95% CI, 0.89-0.97] in the adjusted model; NDVI for 1000 m, OR, 0.79 [95% CI, 0.73-0.84] in the crude model and 0.84 [95% CI, 0.80-0.88] in the adjusted model; SAVI for 100 m, OR, 0.81 [95% CI, 0.74-0.88] in the crude model and 0.90 [95% CI, 0.83-0.95] in the adjusted model; SAVI for 1000 m, OR, 0.69 [95% CI, 0.62-0.77] in the crude model and 0.76 [95% CI, 0.70-0.82] in the adjusted model; *P* < .001 for all) (Table 2). In addition, we observed that the effect estimates increased along with bigger buffers. We categorized NDVI_500-m_ and SAVI_500-m_ into quartiles and found that the estimated ORs gradually decreased with greater quartiles of exposure (eTable 3 in the Supplement). We split ADHD symptoms into predominantly inattention, predominantly hyperactivity-impulsivity, and both inattention and hyperactivity-impulsivity and found that the associations for different subsymptoms were similar (eTable 4 in the Supplement). We also individually adjusted the models for maternal smoking and alcohol consumption during pregnancy, breastfeeding, preterm birth, birth weight, and number of siblings and found that the results were consistent with the main analysis (eTable 5 in the Supplement). We repeated the analyses by excluding children with allergic diseases, congenital heart diseases, preterm birth, or low birth weight and found that the effect estimates were similar to those in the main analysis (eTable 6 in the Supplement). We also repeated the analyses using C-ASQ defined ADHD symptoms and again found associations similar to those observed when using the *DSM-IV* criteria (eTable 7 in the Supplement).

### Do Sociodemographic Factors Modify the Association Between Greenness and ADHD Symptoms?

Table 3 summarizes the associations between greenness and ADHD stratified by age, sex, household income, type of home district, and dog ownership. We found that the odds of having symptoms of ADHD were slightly lower among children aged 7 to 17 years (per 0.1-unit greater NDVI_500-m_, OR, 0.85 [95% CI, 0.81-0.89]; per 0.1-unit greater SAVI_500-m_, OR, 0.78 [95% CI, 0.72-0.84]) than among children aged 2 to 6 years (per 0.1-unit greater NDVI_500-m_, OR, 0.94 [95% CI, 0.86-1.03]; per 0.1-unit greater SAVI_500-m_, OR, 0.90 [95% CI, 0.78-1.04]), and among children living in residential areas (per 0.1-unit greater NDVI_500-m_, OR, 0.86 [95% CI, 0.81-0.90]; per 0.1-unit greater SAVI_500-m_, OR, 0.79 [95% CI, 0.73-0.85]) than those living in business or industrial areas (per 0.1-unit greater NDVI_500-m_, OR, 0.93 [95% CI, 0.85-1.02]; per 0.1-unit greater SAVI_500-m_, OR, 0.89 [95% CI, 0.77-1.03]). However, neither interaction was statistically significant; thus, the results should be interpreted with caution. Also, no significant association was observed for sex, household income, or dog ownership.

**Table 3.** Associations of 0.1-Unit Increase in Normalized Difference Vegetation Index and Soil-Adjusted Vegetation Index Within 500 m of School or Kindergarten With ADHD Symptoms, by Selected Characteristics

Subgroup	Children With ADHD/Total Children, No.	OR (95% CI)^a^	*P* Value for Interaction
Normalized difference vegetation index within 500 m of school or kindergarten			
Age, y			
2-6	197/9823	0.94 (0.86-1.03)	.06
7-17	2369/49 931	0.85 (0.81-0.89)	
Sex			
Male	1647/30 260	0.87 (0.83-0.92)	.54
Female	919/29 494	0.85 (0.80-0.91)	
Annual household income, Yuan^b^			
≤30 000	1705/34 629	0.87 (0.82-0.92)	.95
>30 000	861/25 125	0.86 (0.81-0.92)	
Type of home district			
Residential	2206/52 420	0.86 (0.81-0.90)	.08
Industrial or business	360/7334	0.93 (0.85-1.02)	
Dog ownership			
No	2377/56 380	0.86 (0.82-0.90)	.56
Yes	189/3374	0.84 (0.76-0.92)	
Soil-adjusted vegetation index within 500 m of school or kindergarten			
Age, y			
2-6	197/9823	0.90 (0.78-1.04)	.07
7-17	2369/49 931	0.78 (0.72-0.84)	
Sex			
Male	1647/30 260	0.81 (0.74-0.88)	.63
Female	919/29 494	0.79 (0.71-0.87)	
Annual household income, Yuan^b^			
≤30 000	1705/34 629	0.80 (0.73-0.88)	.95
>30 000	861/25 125	0.80 (0.72-0.88)	
Type of home district			
Residential	2206/52 420	0.79 (0.73-0.85)	.12
Industrial or business	360/7334	0.89 (0.77-1.03)	
Dog ownership			
No	2377/56 380	0.80 (0.74-0.86)	.42
Yes	189/3374	0.75 (0.64-0.87)	

Abbreviations: ADHD, attention-deficit/hyperactivity disorder; OR, odds ratio.

^a^ Adjusted for age, sex, education level, household income level, type of home district, and dog ownership.

^b^ As of November 25, 2019, to convert Yuan to US dollars, multiply by 0.14.

## Discussion

In this large cross-sectional study of Chinese children, we investigated the association between greenness surrounding schools or kindergartens and ADHD symptoms and observed that children studying in greener schools or kindergartens had lower odds of having ADHD symptoms. To our knowledge, this is the first study to evaluate greenness and ADHD symptoms in a developing country. This is also one of the few studies focused on the health outcomes of school-based greenness, where children usually spend most of their daytime.

We are aware of only 1 previous cross-sectional study^10^ evaluating the association of ADHD symptoms with school greenness. In that study from Barcelona, Spain, the authors recruited 2111 children aged 7 to 10 years, measured ADHD symptoms using *DSM-IV* score, and assessed school-based greenness using NDVI. They observed that higher NDVI levels were not significantly associated with fewer ADHD symptoms. Our study had several similarities in design, exposure, and outcome assessment, as well as participants’ age. However, the sample size and number of schools in our study were approximately 28- and 3-fold larger than those in the earlier study. Thus, our study had greater statistical power to detect modest associations, and our estimates may be more precise.

Although studies focusing on school-based estimates of greenness are scarce, several have investigated the associations of other greenness indicators with ADHD symptoms. For instance, Amoly et al^10^ detected beneficial associations between residential greenness and ADHD symptoms in Spanish children. Similarly, a cross-sectional study^14^ of 1717 Korean children found that residential greenness was significantly associated with a lower ADHD score. In a cohort study^13^ of 66 823 German children, it was reported that higher greenness levels in large postal areas were associated with lower risk of ADHD incidence. Two observational studies from Germany^11^ and Lithuania^12^ reported that greater proximity to city parks, but not residential greenness, was associated with fewer ADHD symptoms. Additionally, 1 single-blind controlled trial^8^ and 1 cross-sectional study^9^ found that walking or playing in green settings might decrease the severity of ADHD. Although the results of previously published studies and ours are not identical, the collective evidence generally supports a beneficial association between higher greenness levels and reduced ADHD or ADHD symptoms.

Several mechanisms might explain the association between greenness and ADHD. The biophilia hypothesis suggests that human beings are innately attracted to nature,^37^ and, thus, contact with the natural world is postulated to be beneficial for children’s brain development and attention restoration.^38^ A recent study^39^ of Barcelona schoolchildren provided strong evidence for this hypothesis by evaluating associations between long-term exposure to residential greenness and regional differences in brain volume. They found that greenness exposure was associated with improvements in brain regions associated with better working memory and attentiveness. Higher levels of ambient air pollution have been adversely associated with neurodevelopment as well as ADHD symptoms,^40,41^ and green space has been suggested to reduce levels of air pollutants.^6^ Similarly, exposure to higher ambient noise levels increased ADHD symptoms in children.^42^ The ability of greenness to reduce noise^6,43^ may, therefore, partially explain the beneficial association between greenness and ADHD symptoms in our study. Populations living in greener areas tend to engage in physical activity more frequently,^6^ and sufficient physical activity may improve cognitive development and reduce ADHD symptoms.^44^ Furthermore, failing immunoregulation associated with reduced exposure to macroorganisms and microorganisms is thought to have adverse outcomes on brain development, whereas greenness has the ability to enrich immunoregulation-inducing microbial input from the environment.^45^ This might be another pathway driving our observed association between greenness and ADHD symptoms.

### Implications

As one of the most common neurobehavioral conditions in childhood, ADHD affects approximately 7.2% of youth worldwide and the rate is still increasing in some populations.^1^ Attention-deficit/hyperactivity disorder impairs many areas of children’s lives, including social functioning, academic performance, and overall quality of life, which ultimately lead to major health, social, and economic burdens.^3,4^ Intervention strategies are, therefore, needed to address this pandemic. Our results suggest that attending schools or kindergartens with higher levels of surrounding greenness is associated with lower odds of ADHD symptoms. Given that attention is a critical prerequisite for learning, greenness in school settings may be of great public health significance. Our findings, therefore, are relevant to policy makers and health care authorities for translating evidence into feasible and achievable targeted interventions (eg, planning for green spaces around schools and kindergartens) to mitigate the burden of ADHD in children.

### Strengths and Limitations

This study has several strengths. First, it includes a large population-based sample, covering 94 schools and kindergartens in 7 Chinese cities. Second, China’s school policy restricts student admission according to geographical boundaries, which prevents students from attending transregional schools. In this study, the distance between school and home was short (the mean distance from home to school was 1 km; data not shown); thus, the school-based greenness in larger buffer exposures may also capture children’s greenness exposure near home, and this was further supported by our findings that associations were greater for larger buffers of greenness metrics. Third, we incorporated a set of individual-, family-, and neighborhood-level covariates into the models to control for potential confounding. Fourth, we used 2 exposure metrics and 3 different buffers for green space, as well as 2 different instruments, for assessing ADHD symptoms. In addition, we performed a series of sensitivity analyses to demonstrate that our results were robust.

This study also has some limitations. First, the cross-sectional design did not allow us to identify a temporal sequence between greenness and ADHD symptoms. However, the possibility of reverse causality—that is, the possibility that children with ADHD symptoms may choose a school with less green spaces—is low. Second, we did not have access to personal addresses, but rather school or kindergarten addresses, which means that we only had 94 unique exposure data points for the 59 754 participants. This method might have introduced exposure misclassification. However, the misclassification was likely nondifferential, which often, but not always, biases the results toward null.^46^ Thus, effect estimates may have been greater if we had captured individual exposures. Third, we used satellite image–based NDVI and SAVI to assess greenness exposure; these indices provide information on the general level of vegetation but cannot distinguish between structure, type, and quality of greenness. This prevents us from identifying the specific aspects of green space most closely associated with ADHD symptoms. Fourth, information on covariates and outcomes was collected using questionnaires; thus, a recall bias is possible. Fifth, although we have controlled for a rich set of confounding covariates, several important covariates were still unavailable (eg, meteorological factors and walkability). Thus, the potential for unmeasured confounding is possible. Sixth, ADHD symptoms were measured using questionnaires completed only by parents and guardians and were not clinically verified. In addition, we did not ask whether the children had received a diagnosis of ADHD or were receiving medication. Furthermore, the *DSM-IV* criteria are intended for children aged 6 years or older, but we included children aged 2 to 6 years. Thus, we may have misclassified some children, although we used 2 different validated instruments (*DSM-IV* and C-ASQ) to detect ADHD symptoms and found that the results were consistent.

## Conclusions

This study suggests that greater levels of greenness near schools or kindergartens are associated with lower odds of ADHD symptoms in Chinese children. Our results may be useful for policy makers and health care professionals for developing strategies to mitigate the ADHD burden in China. Well-designed longitudinal epidemiological studies and mechanistic studies are warranted in the future.

## Notes

Supplement. **eTable 1.** Summary of Published Human Studies on Greenness and Attention-Deficit/Hyperactivity Disorder (ADHD) or ADHD Symptoms **eTable 2.** Distributions and Intercorrelations for Greenness Metrics **eTable 3.** Associations Between Quantile Greenness Metrics and Attention-Deficit/Hyperactivity Disorder (ADHD) Symptoms **eTable 4. **Associations Between per 0.1-Unit Greater Greenness Metrics and Subtype of Attention-Deficit/Hyperactivity Disorder (ADHD) Symptoms **eTable 5. **Associations Between per 0.1-Unit Greater Greenness Metrics and Attention-Deficit/Hyperactivity Disorder (ADHD) Symptoms After Additional Adjustment for Smoking and Drinking During Pregnancy, Breastfeeding, Preterm Birth, Birth Weight, and Number of Siblings at Birth **eTable 6. **Associations Between per 0.1-Unit Greater Greenness Metrics and Attention-Deficit/Hyperactivity Disorder (ADHD) Symptoms After Excluding Children With Existing Disorders **eTable 7. **Associations of per 0.1-Unit Greater Greenness Metrics With Attention-Deficit/Hyperactivity Disorder (ADHD) Symptoms Measured by C-ASQ Score **eFigure.** Directed Acyclic Graph for the Association Between Greenness and Attention-Deficit/Hyperactivity Disorder (ADHD) Symptoms, Created With the Help of Dagitty.net
