# Supplementary material for: Association Between Greenness Surrounding Schools and Kindergartens and Attention-Deficit/Hyperactivity Disorder in Children in China
Source: JAMA Netw Open. 2019 Dec 18;2(12):e1917862. doi: 10.1001/jamanetworkopen.2019.17862 (PMC6991306; doi:10.1001/jamanetworkopen.2019.17862)
Supplement: Supplement. — eTable 1. Summary of Published Human Studies on Greenness and Attention-Deficit/Hyperactivity Disorder (ADHD) or ADHD Symptoms eTable 2. Distributions and Intercorrelations for Greenness Metrics eTable 3. Associations Between Quantile Greenness Metrics and Attention-Deficit/Hyperactivity Disorder (ADHD) Symptoms eTable 4. Associations Between per 0.1-Unit Greater Greenness Metrics and Subtype of Attention-Deficit/Hyperactivity Disorder (ADHD) Symptoms eTable 5. Associations Between per 0.1-Unit Greater Greenness Metrics and Attention-Deficit/Hyperactivity Disorder (ADHD) Symptoms After Additional Adjustment for Smoking and Drinking During Pregnancy, Breastfeeding, Preterm Birth, Birth Weight, and Number of Siblings at Birth eTable 6. Associations Between per 0.1-Unit Greater Greenness Metrics and Attention-Deficit/Hyperactivity Disorder (ADHD) Symptoms After Excluding Children With Existing Disorders eTable 7. Associations of per 0.1-Unit Greater Greenness Metrics With Attention-Deficit/Hyperactivity Disorder (ADHD) Symptoms Measured by C-ASQ Score eFigure. Directed Acyclic Graph for the Association Between Greenness and Attention-Deficit/Hyperactivity Disorder (ADHD) Symptoms, Created With the Help of Dagitty.net [file jamanetwopen-2-e1917862-s001.pdf]

Supplementary Online Content

Yang B-Y, Zeng X-W, Markevych I, et al. Association between greenness surrounding schools and kindergartens and attention-deficit/hyperactivity disorder in children in China. *JAMA Netw Open.* 2019;2(12):e1917862. doi:10.1001/jamanetworkopen.2019.17862

eTable 1. Summary of Published Human Studies on Greenness and Attention-Deficit/Hyperactivity Disorder (ADHD) or ADHD Symptoms
eTable 2. Distributions and Intercorrelations for Greenness Metrics
eTable 3. Associations Between Quantile Greenness Metrics and Attention-Deficit/Hyperactivity Disorder (ADHD) Symptoms
eTable 4. Associations Between per 0.1-Unit Greater Greenness Metrics and Subtype of Attention-Deficit/Hyperactivity Disorder (ADHD) Symptoms
eTable 5. Associations Between per 0.1-Unit Greater Greenness Metrics and Attention-Deficit/Hyperactivity Disorder (ADHD) Symptoms After Additional Adjustment for Smoking and Drinking During Pregnancy, Breastfeeding, Preterm Birth, Birth Weight, and Number of Siblings at Birth
eTable 6. Associations Between per 0.1-Unit Greater Greenness Metrics and Attention-Deficit/Hyperactivity Disorder (ADHD) Symptoms After Excluding Children With Existing Disorders
eTable 7. Associations of per 0.1-Unit Greater Greenness Metrics With Attention-Deficit/Hyperactivity Disorder (ADHD) Symptoms Measured by C-ASQ Score
eFigure. Directed Acyclic Graph for the Association Between Greenness and Attention-Deficit/Hyperactivity Disorder (ADHD) Symptoms, Created With the Help of Dagitty.net

This supplementary material has been provided by the authors to give readers additional information about their work.

© 2019 Yang B-Y et al. *JAMA Network Open.*

eTable 1. Summary of Published Human Studies on Greenness and Attention-Deficit/Hyperactivity Disorder (ADHD) or ADHD Symptoms

Authors, publication year [reference]	Study design	Study participants	Outcome metrics	Exposure metrics	Main findings
Taylor and Kuo, 2009	Single-blind controlled trials	17 children aged 7-12 years with diagnosed ADHD in the United States	Concentration measured using Digit Span Backwards	Walk in park, downtown, or neighborhood.	Walking in park was associated with better concentration of ADHD children compared to children walking in downtown or neighborhood.
Kuo and Taylor, 2004	Cross-sectional study	452 children aged 5-18 years with diagnosed ADHD in the United States	Diagnostic and Statistical Manual of Mental Disorders, Fourth Edition (ADHD/DSM-IV)	Forty-nine survey items representing the broad range of activities, physical settings, and social contexts children experience outside of school	Greener outdoor settings reduce presence of ADHD symptoms in children. Sex, age, income, community type, and geographical region did not modify the association.
Amoly et al., 2014	Cross-sectional study	2111 schoolchildren (7-10 years of age) from Barcelona	ADHD evaluated by Strengths and Difficulties Questionnaires (SDQ) and Diagnostic and Statistical Manual of Mental Disorders, Fourth Edition (ADHD/DSM-IV)	Time spent playing in green spaces; residential surrounding greenness (NDVI), and residential proximity to a major green space.	There exist significant and inverse associations of ADHD symptoms scores with residential greenness, but not with residential proximity to major green space. School greenness was not associated with ADHD symptoms.
Markevych et al. 2014	Cross-sectional study	1932 Children and adolescents (10 years of age) from two German cohorts	ADHD symptoms assessed using the SDQ	Distance to the nearest urban green space	The distance between residence and the nearest urban green space was positively associated with the odds of hyperactivity/inattention symptoms, and sex modified the association.
Balseviciene et al. 2014	Cohort study	1468 mothers and their 4- to 6- year-old children living in the city of Kaunas, Lithuania	Emotional and behavioral problems assessed using the SDQ (ADHD symptoms)	Proximity to city parks and residential greenness	Farther distance from city parks was associated with hyperactivity in children whose mothers had a lower education levels, but not in children whose mothers had a higher education levels. No significant association was observed or NDVI and

© 2019 Yang B-Y et al. *JAMA Network Open.*

					hyperactivity.
Dadvand et al. 2017	Cohort study	Two prospective birth cohorts from Spain during 2003-2013. Participants were followed at pregnancy, 2y, 4y, or 5y, and 7 y of age. The sample size was 978 for Attentional Network Test or 888 for Conners' Kiddie Continuous Test	Attention assessed using Conners Kiddie Continuous Performance Test and Attentional Network Task [sic]	Residential greenness was assessed using two satellite-based indices of greenness: NDVI and vegetation continuous fields	Higher residential greenness levels were associated with better scores on tests of attention.
Markevych et al. 2018	Cohort study	66,823 children (aged 10-14 years) residing in Germany since 2005	ADHD incidence was diagnosed by at least one outpatient ICD-10-GM F90 by a child/adolescent psychiatrist, neuropediatrician, or psychotherapist	NDVI	An increase of 0.1-unit in NDVI decreased the relative risk of ADHD by a factor of 0.82 (95% confidence interval: 0.68-0.98)
Lee et al., 2019	Cross-sectional study	1817 Korean children (aged 7-17 years) and adolescents from 2012 to 2013	ADHD was assessed using the Child Behavior Checklist	Residential greenness evaluated using modified soil-adjusted vegetation index	A significantly lower score on ADHD was associated with highly modified soil-adjusted vegetation index levels.

Abbreviations: ADHD, attention deficit hyperactivity disorder; SDQ, Strengths and Difficulties Questionnaires; ADHD/DSM-IV, ADHD/Diagnostic and Statistical Manual of Mental Disorders, Fourth Edition.

© 2019 Yang B-Y et al. *JAMA Network Open.*

eTable 2. Distributions and Intercorrelations for Greenness Metrics

	Statistics					Spearman Correlation Coefficient					
Greenness	Median (IQR)	Min	Max	Mean (SD)		$NDVI_{100\text{-}m}$	$NDVI_{500\text{-}m}$	$NDVI_{1000\text{-}m}$	$SAVI_{100\text{-}m}$	$SAVI_{500\text{-}m}$	$SAVI_{1000\text{-}m}$
$NDVI_{100\text{-}m}$	0.27 (0.21 to 0.38)	-0.13	0.82	0.31 (0.16)		1	0.83[a]	0.70[a]	0.99[a]	0.83[a]	0.70[a]
$NDVI_{500\text{-}m}$	0.29 (0.23 to 0.38)	-0.09	0.77	0.31 (0.14)			1	0.93[a]	0.81[a]	0.99[a]	0.92[a]
$NDVI_{1000\text{-}m}$	0.28 (0.23 to 0.36)	0.05	0.68	0.31 (0.12)				1	0.70[a]	0.93[a]	0.99[a]
$SAVI_{100\text{-}m}$	0.15 (0.11 to 0.22)	-0.02	0.50	0.18 (0.10)					1	0.83[a]	0.72[a]
$SAVI_{500\text{-}m}$	0.16 (0.12 to 0.21)	0.00	0.47	0.18 (0.09)						1	0.94[a]
$SAVI_{1000\text{-}m}$	0.16 (0.13 to 0.20)	0.05	0.42	0.18 (0.08)							1

Abbreviations: IQR, interquartile range; Min, minimum; Max, maximum; NDVI, normalized difference vegetation index; SAVI, soil adjusted vegetation index; SD, standard deviation.
[a]$P < 0.05$ for correlation.

© 2019 Yang B-Y et al. *JAMA Network Open.*

eTable 3. Associations Between Quantile Greenness Metrics and Attention-Deficit/Hyperactivity Disorder (ADHD) Symptoms (n = 59 754)

	Adjusted Model[a]		
Greenness Metrics	OR (95% CI)	*P* Value	*P* Value for Trend
$NDVI_{500\text{-}m}$			< .001
Q_1 (<0.23)	Ref.		
Q_2 (0.23-0.29)	0.89 (0.80 to 0.99)	.03	
Q_3 (0.30-0.38)	0.68 (0.60 to 0.76)	< .001	
Q_4 (>0.38)	0.63 (0.55 to 0.71)	< .001	
$SAVI_{500\text{-}m}$			< .001
Q_1 (<0.12)	Ref.		
Q_2 (0.12-0.16)	0.92 (0.83 to 1.02)	.11	
Q_3 (0.17-0.21)	0.70 (0.63 to 0.78)	< .001	
Q_4 (>0.21)	0.64 (0.57 to 0.73)	< .001	

Abbreviations: ADHD, attention deficit hyperactivity disorder; CI, confidence interval; NDVI, normalized difference vegetation index; OR, odds ratio; SAVI, soil adjusted vegetation index.
[a]Adjusted for age, gender, parental education level, household income level, type of home district, and dog ownership.

© 2019 Yang B-Y et al. *JAMA Network Open.*

eTable 4. Associations Between per 0.1-Unit Greater Greenness Metrics and Subtype of Attention-Deficit/Hyperactivity Disorder (ADHD) Symptoms (N = 59 754)

	Adjusted Model[a]	
ADHD subtype	OR (95% CI)	*P* Value
ADHD-HI		
$NDVI_{500\text{-}m}$	0.87 (0.80 to 0.95)	.002
$SAVI_{500\text{-}m}$	0.81 (0.71 to 0.93)	.003
ADHD-I		
$NDVI_{500\text{-}m}$	0.87 (0.83 to 0.91)	<.001
$SAVI_{500\text{-}m}$	0.80 (0.74 to 0.87)	<.001
ADHD-C		
$NDVI_{500\text{-}m}$	0.88 (0.79 to 0.97)	.01
$SAVI_{500\text{-}m}$	0.82 (0.70 to 0.97)	.02

Abbreviations: ADHD, attention deficit hyperactivity disorder; ADHD-C, ADHD symptoms including both inattention and hyperactivity-impulsivity symptoms; ADHD-HI, ADHD symptoms including predominantly hyperactivity-impulsivity; ADHD-I, symptoms including predominantly inattention; CI, confidence interval; NDVI, normalized difference vegetation index; OR, odds ratio; SAVI, soil adjusted vegetation index.

[a]Adjusted for age, gender, parental education level, household income level, type of home district, and dog ownership.

© 2019 Yang B-Y et al. *JAMA Network Open.*

eTable 5. Associations Between per 0.1-Unit Greater Greenness Metrics and Attention-Deficit/Hyperactivity Disorder (ADHD) Symptoms After Additional Adjustment for Smoking and Drinking During Pregnancy, Breastfeeding, Preterm Birth, Birth Weight, and Number of Siblings at Birth (N = 59 754)

Models	OR (95% CI)	*P* Value
$NDVI_{500\text{-}m}$		
Main model[a] + smoking during pregnancy	0.87 (0.83 to 0.91)	<.001
Main model[a] + drinking during pregnancy	0.87 (0.83 to 0.91)	<.001
Main model[a] + breastfeeding	0.87 (0.83 to 0.91)	<.001
Main model[a] + preterm birth	0.87 (0.83 to 0.91)	<.001
Main model[a] + birth weight	0.87 (0.83 to 0.91)	<.001
Main model[a] + number of siblings	0.86 (0.83 to 0.90)	<.001
$SAVI_{500\text{-}m}$		
Main model[a] + smoking during pregnancy	0.80 (0.74 to 0.86)	<.001
Main model[a] + drinking during pregnancy	0.80 (0.74 to 0.86)	<.001
Main model[a] + breastfeeding	0.80 (0.74 to 0.86)	<.001
Main model[a] + preterm birth	0.80 (0.75 to 0.86)	<.001
Main model[a] + birth weight	0.80 (0.75 to 0.86)	<.001
Main model[a] + number of siblings	0.80 (0.74 to 0.86)	<.001

Abbreviations: ADHD, attention deficit hyperactivity disorder; CI, confidence interval; NDVI, normalized difference vegetation index; OR, odds ratio; SAVI, soil adjusted vegetation index.
[a]Adjusted for age, gender, parental education level, household income level, type of home district, and dog ownership.

© 2019 Yang B-Y et al. *JAMA Network Open.*

eTable 6. Associations Between per 0.1-Unit Greater Greenness Metrics and Attention-Deficit/Hyperactivity Disorder (ADHD) Symptoms After Excluding Children With Existing Disorders

Models	No.	OR (95% CI)[a]	*P* Value
$NDVI_{500\text{-}m}$			
Excluding children with allergic diseases[b]	48 187	0.86 (0.82 to 0.91)	<.001
Excluding children with congenital heart diseases	59 493	0.86 (0.82 to 0.91)	<.001
Excluding children with preterm birth	56 537	0.86 (0.82 to 0.91)	<.001
Excluding children with low birth weight	57 567	0.87 (0.83 to 0.91)	<.001
$SAVI_{500\text{-}m}$			
Excluding children with allergic diseases[b]	48 187	0.80 (0.74 to 0.86)	<.001
Excluding children with congenital heart diseases	59 493	0.80 (0.74 to 0.86)	.002
Excluding children with preterm birth	56 537	0.80 (0.74 to 0.86)	<.001
Excluding children with low birth weight	57 567	0.80 (0.74 to 0.87)	<.001

Abbreviations: ADHD, attention deficit hyperactivity disorder; CI, confidence interval; NDVI, normalized difference vegetation index; OR, odds ratio; SAVI, soil adjusted vegetation index.
[a]Adjusted for age, gender, parental education level, household income level, type of home district, and dog ownership.
[b]Allergic diseases included asthma, eczema, and allergic conjunctivitis.

© 2019 Yang B-Y et al. *JAMA Network Open.*

eTable 7. Associations of per 0.1-Unit Greater Greenness Metrics With Attention-Deficit/Hyperactivity Disorder (ADHD) Symptoms Measured by C-ASQ Score (N = 59 754)

Greenness Metrics	Adjusted Model[a]	
	OR (95% CI)	*P* Value
$NDVI_{500\text{-}m}$	0.85 (0.79 to 0.90)	<. 001
$SAVI_{500\text{-}m}$	0.77 (0.70 to 0.86)	<. 001

Abbreviations: ADHD, attention deficit hyperactivity disorder; C-ASQ, the Conners Abbreviated Symptom Questionnaire; CI, confidence interval; NDVI, normalized difference vegetation index; OR, odds ratio; SAVI, soil adjusted vegetation index.
[a]Adjusted for age, gender, parental education level, household income level, type of district, and dog ownership.

© 2019 Yang B-Y et al. *JAMA Network Open.*

eFigure. Directed Acyclic Graph for the Association Between Greenness and Attention-Deficit/Hyperactivity Disorder (ADHD) Symptoms, Created With the Help of Dagitty.net (www.dagitty.net)

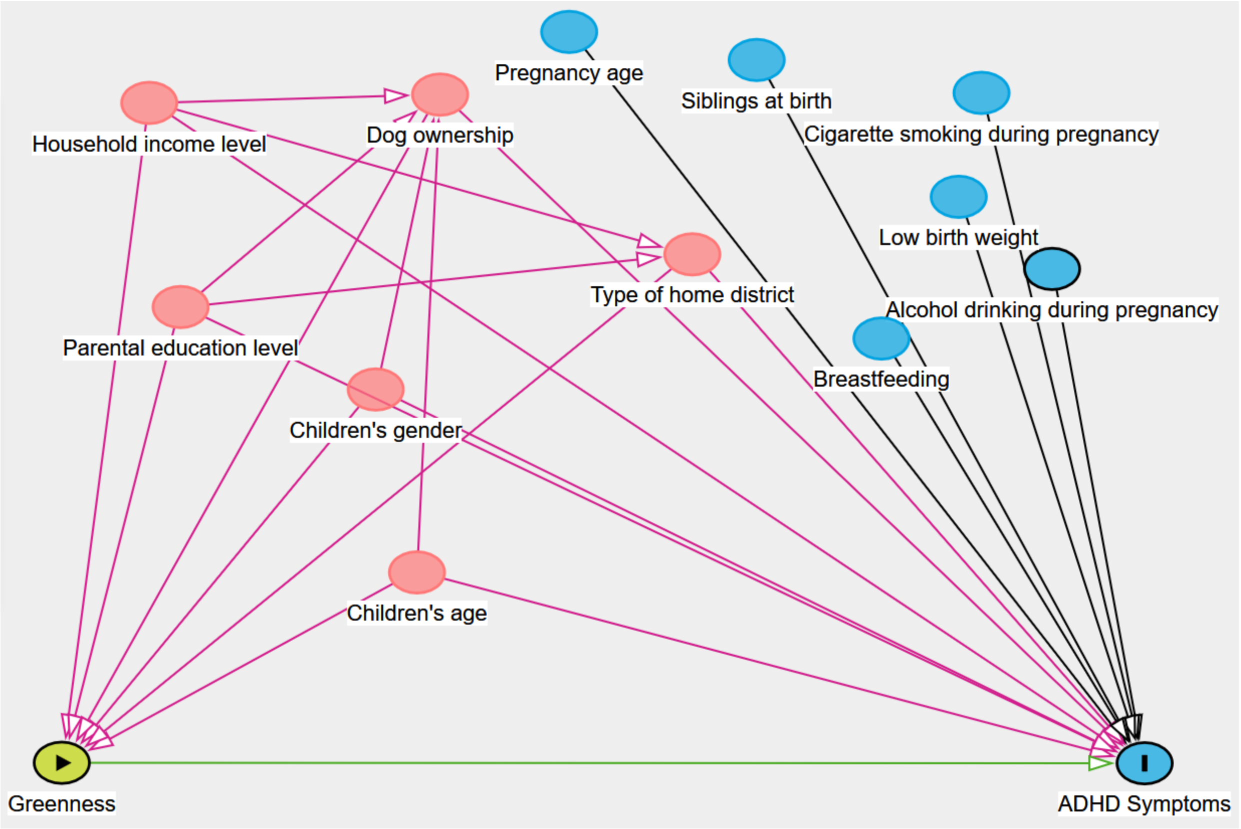

© 2019 Yang B-Y et al. *JAMA Network Open.*
